# Assessment of Satisfaction with Health Services among Prisoners—Descriptive Study

**DOI:** 10.3390/healthcare10030548

**Published:** 2022-03-16

**Authors:** Anna Rogalska, Kamil Barański, Żaneta Rachwaniec-Szczecińska, Tomasz Holecki, Monika Bąk-Sosnowska

**Affiliations:** 1 Department of Health Economics and Health Management, Faculty of Health Sciences in Bytom, Medical University of Silesia, 40-055 Katowice, Poland; tholecki@sum.edu.pl; 2 Department of Epidemiology, Faculty of Medical Sciences, Medical University of Silesia, 40-055 Katowice, Poland; kbaranski@sum.edu.pl; 3 Department of Psychology, School of Health Sciences in Katowice, Medical University of Silesia, 40-055 Katowice, Poland; zaneta.szczecinska@sum.edu.pl (Ż.R.-S.); monika.bak-sosnowska@sum.edu.pl (M.B.-S.)

**Keywords:** healthcare in prison, the health of prisoners, access to healthcare services in prison, assessment of satisfaction with health services in prison

## Abstract

Aim: The aim of the study was to assess the access of prisoners to healthcare services, as well as the level of satisfaction with the provided services and health assessment among prisoners. Methods: The research was conducted in one of the penitentiary centers in Poland among people jailed between 1 January to 31 January 2020. The response rate of the self-administrated questionnaire was 52.05% (469/901) participants. There were 389 men and 77 women. Results: Prisoners assessed access to health services including GP doctors, specialist doctors, dentists, and hospitals in 3 categories: “bad” ranged: 27.03–67.60%; “medium” ranged: 22.54–53.57%; “good” ranged: 7.02–33.96% depending on the type of arrest, but no statistical significance was demonstrated. Satisfaction with the health services defined as “bad” ranged: 25.00–61.11%; “medium” ranged: 18.97–55.56%; “good” ranged: 5.56–34.62% depending on the type of arrest but no statistical significance was demonstrated. Of 469 prisoners, 215 prisoners (45.84%) declared no addictions. The frequency of addiction does not differ depending on the place/type of punishment served (*p* = 0.9). In turn, 317 prisoners (68%) declared no chronic diseases. Conclusions: Most of the prisoners described access to health services as “bad”, except female prisoners from a semi-open facility. In turn, satisfaction with healthcare services was most often assessed as “bad”, except for temporarily arrested men and female prisoners from a semi-open facility.

## 1. Introduction

The number of prisoners is increasing worldwide, however, there are differences in variation between continents and within continents. According to statistical data (from 2021), the most prisoners were in the United States (almost 2.1 million people in prisons), followed by China, Brazil, India, and the Russian Federation [1]. On the other hand, the number of prisoners per 100,000 population in the world varies greatly from 37 in Japan, 70 in Germany, 91 in Austria, in Estonia, 190 in Poland, 253 in Georgia, 347 in Turkey, Rwanda 580, and 629 in the United States [2]. According to United Nations estimates, the world prison population rate for a national population level is 140 per 100,000 [3].

Although the functioning of prison systems varies around the world, in many countries similar problems can be found in the prison system, including overpopulation, poor health of prisoners, inactivity, violence and abuse, addictions, and limited access to healthcare which pose challenges to public health. Access to healthcare is critical to the functioning of healthcare systems around the world. For healthcare, access can be defined as the possibility or ease with which consumers can obtain appropriate services commensurate with their needs [4,5]. Access to healthcare in prisons is important for reducing inequalities among marginalized groups [6]. Sanhueza suggests that access to healthcare is related to other aspects of prison life, such as the composition of the prison population (gender), some material aspects of prisons (infrastructure, type of facility), and even some relational aspects (levels of ill-treatment/abuse) [7]. Problems with access to healthcare in prisons occur in various countries. Access to healthcare may refer to various aspects, not only of treatment but also of prevention [7,8].

### 1.1. Health of Prisoners

Prisoners are persons in detention who have been convicted or are currently under prosecution, trial, or sentence for unlawful offenses [6]. An important aspect from the point of view of public health is the way healthcare works in prisons. Worldwide, persons in prisons have a higher incidence of both acute and chronic disease than the general population [9]. Prison populations contain a high prevalence of people with serious and often life-threatening conditions [10]. Moreover, this group often reports a higher incidence of infectious diseases and chronic conditions compared to the non-institutionalized population [11]. Prisoners are recognized as vulnerable to poor health and lifestyle choices, as well as accelerated aging processes [12]. Healthcare provision for prisoners has several common features. The state is responsible for providing total healthcare, and, on average, detainees have a higher morbidity rate, which implies greater healthcare needs than non-detained persons [13,14]. To analyze the health condition of prisoners, the literature takes into account, inter alia, indicators, such as BMI, the occurrence of chronic diseases, and addictions [15,16,17].

### 1.2. Organization of Health Care in Polish Prisons

The Polish systems of executing the sentence of imprisonment were divided into various types of penitentiary units and types of classification of convicts. This state of affairs undoubtedly makes it necessary to adjust the healthcare organization of prisoners depending on the conditions of their isolation.

According to Polish law, prisons can be divided into the following types: (a) closed, (b) semi-open, and (c) open. Standards for healthcare in penitentiary units have been established in the Executive Penal Code, the Act of 9 April 2010 on the Prison Service, and regulations. The Polish healthcare system is mainly based on health insurance. Under the Polish public healthcare system, people covered by health insurance are entitled to health services. However, prisoners are provided with health services free of charge, regardless of their health insurance rights. Additionally, they are financed entirely from the state budget [18]. In the Polish legal system, the convicted person is provided with free health services, medicines, and sanitary articles. On the other hand, in the case of citizens who are not in prison, drugs are given to recipients due to their clinical condition: free of charge, or for a lump sum, or a fee of 30% or 50% of their financing limit [19]. Moreover, in prison, the relationship between patients and medical personnel is not based on free choice [20]. The initial state of health of people in prison can affect the expenses for the entire prison. A prison service report shows that in 2020 258,749 outpatient examinations were performed, of which 61.66% were realized in the non-prison health service. On the other hand, physiotherapeutic procedures (total number of services 42,125) in non-prison healthcare were performed to a small extent—0.14%. The total average expenses for the maintenance of 1 prisoner in 2020 in Poland amounted to PLN 4093.75/month (EUR 895.71—as of 20 October 2021) [21]. In the Polish healthcare system, healthcare among prisoners differs in the way it is organized compared to the general population. According to the Criminal Code, a prisoner serving a sentence of imprisonment is not entitled to choose: (a) an outpatient physician and nurses with an infirmary; (b) primary healthcare physician, primary healthcare nurse, and primary healthcare midwife, and (c) outpatient healthcare providers, dentists, and hospitals [22]. Taking into account factors such as the limited location of prisoners, as well as the number of service providers providing medical services to persons deprived of liberty, the possibility of choosing a service provider would entail an additional logistical, organizational, and financial burden. On the other hand, statutory access to free medicines, prostheses, orthopedic devices, and aids for prisoners [22] (to which insured persons have to pay extra out of pocket) is necessary for their treatment process.

Taking into consideration the disproportions between the functioning of the healthcare system in the prison population and beyond, the study aimed to assess the availability of healthcare services, as well as the level of satisfaction with the provided health services among prisoners and health assessment among prisoners.

## 2. Material and Methods

### 2.1. Study Design

The research was conducted in an ordinary criminal detention facility in Poland among people staying there from 1 January to 31 January 2020. The questionnaire was distributed by employees of the prison. The survey was anonymous and voluntary, no data were collected based on which the respondents could be identified. The inclusion criteria for the study were as follows: (a) adults, (b) being in prison independently on the type of arrest. The criteria for exclusion from the study included: (a) convicted of serious crimes; (b) mentally diseased people; (c) minors. The questionnaire included socio-demographic questions as well as questions on data access assessment of medical service satisfaction, as well as questions about chronic diseases, medications, addiction, and healthcare use.

Out of 901 prisoners residing in a penitentiary unit in the above-mentioned period, a return from the questionnaires obtained with the completion of all answers was obtained from 469 prisoners.

### 2.2. Statistical Analysis

The qualitative variables were expressed as frequency (n) and percentage (%). The relationship between qualitative variables was assessed by the Chi-square test or Fisher test, as appropriate. The level of significance in statistical analysis was set at *p* < 0.05 value. All analyses were performed using TIBCO (TIBCO Software Inc., Palo Alto, CA, USA) Statistica version 13.

Each person was assigned the following variables, such as gender and age; place of residence (city, village), weight, height, education, having a partner; having children; professional situation, believer/non-believer; disease entities. BMI was calculated for the participant and their nutritional status was classified according to the standards of the World Health Organization (WHO).

## 3. Results

### 3.1. Demographics, Description of the Study Population

In the conducted study, the majority were men, who comprised slightly more than 83% (n = 389) of all respondents, while slightly more than 16% (n = 77) were women. The smallest group were prisoners with higher education 7.5% (n = 34), the next group were prisoners with primary education 26% (n = 116), another group was represented by prisoners with secondary education 29% (n = 131), while the largest group was prisoners with vocational education 37% (n = 169).

The mean age in the entire study group was 39.1 ± 11.8. In the group of first-time convicts, the average age was 37.5 ± 11.0, among temporarily arrested 40.8 ± 15.2, convicted recidivists imprisoned in a semi-open institution 40.4 ± 9.6, in a closed unit the average age was 40.8 ± 13.3, and for women imprisoned in a semi-open institution 38.4 ± 10.5, the average age of women temporarily imprisoned was 39.3 ± 12.3. These groups did not differ in terms of age.

Among all prisoners, a trend can be seen that the percentage of prisoners is decreasing due to the number of children born annually. The study showed that the higher the birthrate, the fewer detainees.

### 3.2. Health and Health Behaviors of Prisoners

Based on the height and weight data provided, the BMI was calculated (n = 455). The average BMI was 23.05 ± 3.94 kg/m^2^. Almost half, 215 prisoners (45.84%), declared no addictions, while 121 (25.8%) respondents declared that they were smokers. The frequency of addiction does not differ depending on the place/type of punishment served (*p* = 0.9). Among all prisoners, 68% (n = 317) declared no chronic diseases. A similar trend can be seen (*p* < 000.1) when analyzing the subgroups. People most often suffering from diseases are persons temporarily arrested without a sentence, in this group, there were 49% (n = 26) of such prisoners. A similar trend can be observed in the field of drugs used (*p* = 0.001).

### 3.3. Subjective Assessment of Access to Health Care among Prisoners

The subjective assessment of the access to different levels of healthcare was assessed on a scale of the “bad”, “medium”, and “good”.

Prisoner assessment of access to the medical services of a GP at a “bad” level was declared by 47.98% (min. 30.19–max. 59.09), “medium” 36.95% (min. 25.76–max. 50.00), “good” 15.07% (min.6.25–max.33.96) for each type of health service but no statistical significance was demonstrated.

Assessment of access to a dentist’s services described as “bad” 37.59% (min. 27.03–max. 56.16), “medium” 39.78% (min. 26.03–max. 53.57), “good” 22.64% (min.14.29–max 35.85) for each type of health service.

Prisoner assessment of access to the medical services of specialist care at a “bad” level was declared by 50.14% (min. 39.62–max. 63.77), “medium” 36.53% (min. 26.09–max. 52.78), “good” 13.33% (min. 7.41–max. 28.30) for each type of health service.

Assessment of access to the hospital as “bad” 56.90% (min. 49.06–max. 67.61), “medium” 31.97% (min. 22.54–max. 40.74), “good” 11.12% (min. 7.02–max. 20.75) for each type of health service.

**Figure 1:**
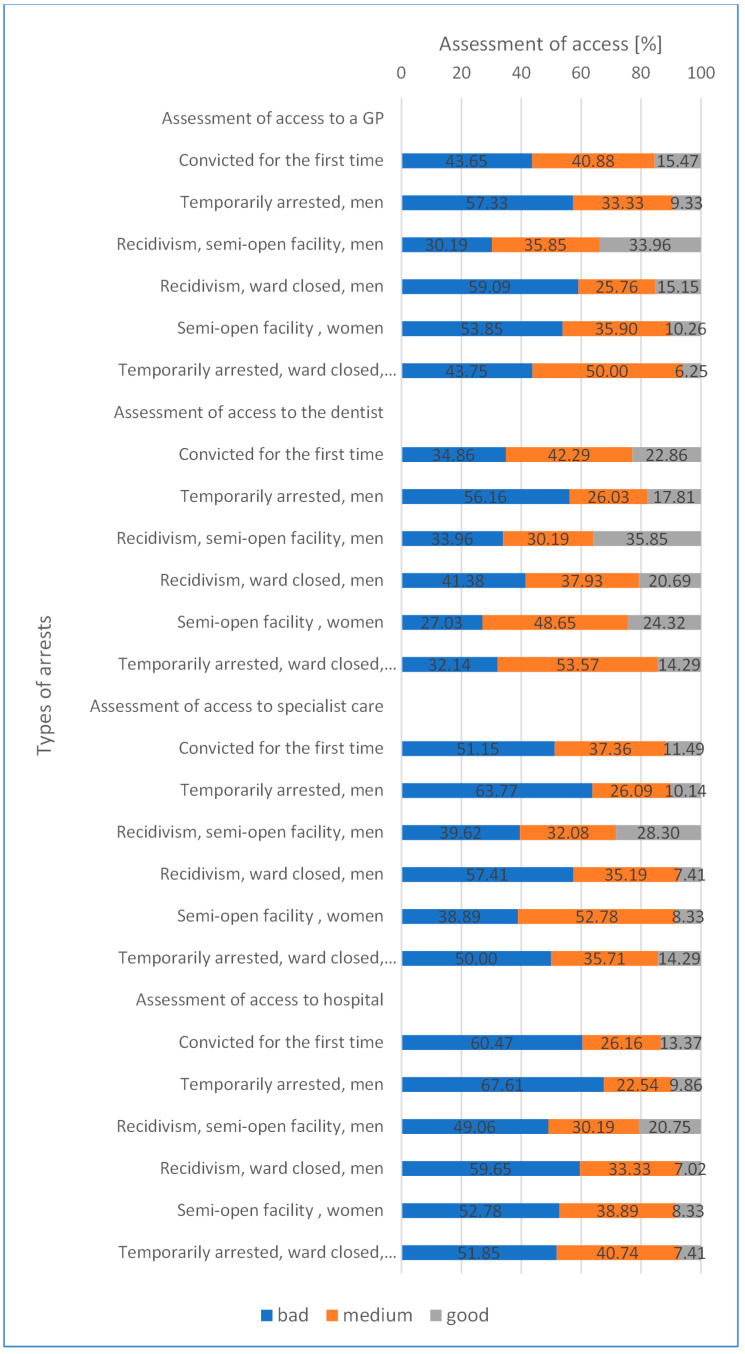
Subjective assessment of access to healthcare among prisoners (“bad”, “medium”, “good”) (test Chi-square).

Average prisoners’ assessment for access to medical care in prison including GP doctor, specialist doctor, dentist, and hospital in 3 categories: “bad” 48.15% (min. 27.03–max. 67.61); “medium” 36.31% (min. 22.54–max. 53.57); “good” 15.54 (min. 7.02–max. 33.96) depending on the type of arrest, but no statistical significance was demonstrated (Figure 1).

### 3.4. Satisfaction with the Health Services and Use of Health Services

Satisfaction with the following medical services was assessed: primary care physician, dentist, specialist physician divided into 3 categories: “bad”, “medium”, “good” by type of arrest.

Prisoner assessed satisfaction with the medical services of a GP at a “bad” level was declared by 51.03% (min. 32.08–max. 61.11), “medium” 33.70% (min. 22.73–max. 39.62), “good” 15.27% (min. 5.56–max. 28.30) depending on the type of arrest, but no statistical significance was demonstrated.

Satisfaction with dentist’s services as assessed as “bad” 40.85% (min. 25.00–max. 58.62), “medium” 34.54% (min. 19.87–max. 55.56), “good” 24.62% (min. 18.06–max. 34.62) for each type of health service but no statistical significance was demonstrated.

**Figure 2:**
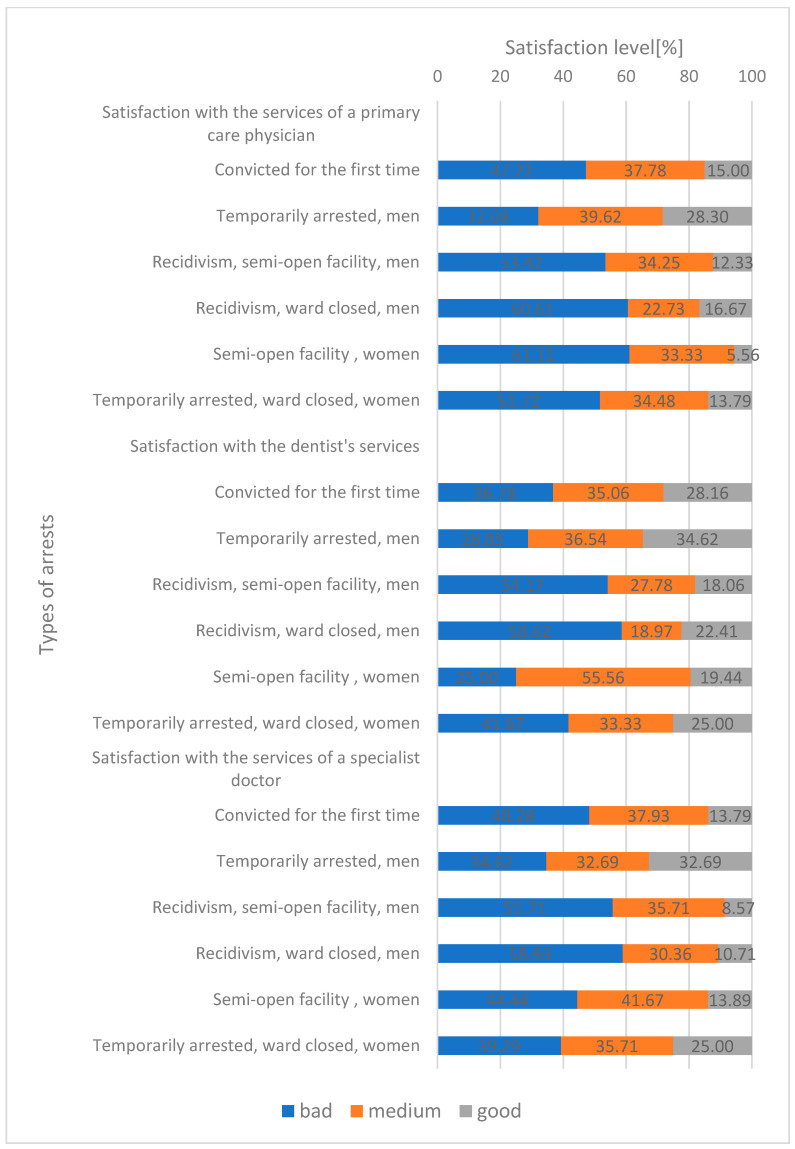
Satisfaction with the health services (“bad”, “medium”, “good”) (test Chi-square).

Prisoners assessed satisfaction with the services of a specialist doctor as “bad” 46.88% (min. 34.62–max. 58.93), “medium” 35.68% (min. 30.36–max. 41.67), “good” 17.44% (min. 8.57–max. 32.69) for each type of health service but no statistical significance was demonstrated (Figure 2).

Average prisoners’ assessment of access to medical care in a prisoner including GP doctor, specialist doctor, dentist services as “bad” 46.25% (min. 25.00–max. 61.11); “medium” 34.64% (min. 18.97–max. 55.56); “good” 19.11 (min. 5.56–max. 34.62) depending on the type of arrest (Figure 2).

In the case of physiotherapeutic procedures, 6.18% (n = 29) of prisoners declared using these services. On the other hand, 17.48% (n = 82) declared that they had used an ambulance. On the other hand, operations during their stay in a penitentiary unit were experienced by 21 respondents (4.48%).

## 4. Discussion

Because in the Polish prison system, prisoners do not have free access to external healthcare services, it seems extremely important to assess both access to and satisfaction with medical services. In this study, the highest assessment of access to medical services at a “good” level at to a GP doctor (33.96%) was shown among in men recidivism semi-open facilities. The highest level of “good” access to a hospital (20.75%) was found in the group of semi-open facilities for recidivism for men. It was similar in the case of access to specialist care (20.30%) and access to a dentist (35.85%). Condon et al. showed that the majority of prisoners stated that their stay in prison was an opportunity to catch up on healthcare and take advantage of the services offered [23]. In contrast, Pettit et al. showed in their study that access to basic healthcare for prisoners was limited [24]. La Cerra et al. concluded that a primary care model can improve adequate health management outcomes in prison settings [25]. Similarly, in the study by Geitonai et al., 46.5% of prisoners declared access to low-level healthcare [26]. In turn, Kouyoumdjian et al. showed rates of all types of healthcare use were significantly higher in prison and on release for those released from prison compared to the general control population [27].

In addition to access to medical services, measuring patient satisfaction is a necessary element in the evaluation of health services in terms of service quality and the responsiveness of the healthcare system [28]. In this study, the “bad” satisfaction with medical services among prisoners ranged from 25% to 61%, depending on the type of services and the type of prisoners. However, in the research on opinions on the healthcare system in Poland conducted in 2020, 62% of the general population were dissatisfied with the healthcare system in Poland in 2020 [29]. Polak et al. showed that only 25% of Poles declared “very good” and “good” satisfaction with healthcare among Poles in 2016 [30]. In turn, Zawisza et al. showed in a study of satisfaction with medical care among the elderly in Poland that only 16% were dissatisfied and satisfaction significantly depended on the waiting time for treatment, the doctor’s respect, obtaining a better explanation of the health condition from the medical team, participation in the decision-making process, the doctor’s sense of interest in the patient’s private matters, and the possibility of choosing a doctor [31].

Referring to the nutritional status examination of prisoners using the BMI index, it was shown that the average was a BMI = 23.05 (SD 3.94), which is in normal weight in contrast to the studies by Baldwin et al., showed that a BMI at imprisonment = 27.4 [32]. Abera et al. have shown that the effect of confinement on the BMI of adult males in certain minority racial/ethnic education groups is variable [33]. Conversely, other researchers showed an increase in body weight or BMI during or after incarceration [34,35,36].

When referring to addictions, the majority (54.96%) of prisoners declared that they had some kind of addiction. In the case of prisoners, 25.8% declared smoking, i.e., less than in the report of the National Health Account of Poles in 2020, which showed that the percentage of active smokers of all tobacco products is 32% [37]. Wand et al. showed that three-quarters of current prisoners in Australia are tobacco smokers [38]. Smoking is a learned habit and a major preventable chronic disease [39].

Analyzing the health of prisoners, 32% reported a chronic disease or were constantly taking medications. In the case of the entire Polish population, it is estimated that 48% of Poles report a chronic disease. Because the prevalence of chronic diseases increases with age, the mean age of prisoners (39.1 ± 11.8) was compared with the Medonet study, where 42% of people in the 35–44 age group suffer from chronic diseases [37]. Wilper et al. showed that among prisoners in the United States (US), 39% of federal prisoners and 42% of state prisoners suffer from a chronic medical condition [11]. Studies by other authors show that chronic diseases are more common in the prison population compared with the general population [9,11,40]. Dealing with health problems in prisons can be difficult because the primary function of prisons is to correct and punish prisoners, which, in turn, may interfere with the quality of health [41,42].

Our study had some limitations. In this study, only one prison was investigated, and we did not evaluate cause–effect factors of healthcare satisfaction.

## 5. Conclusions

Subjective assessment of access to healthcare in prisoners was assessed at a low level, similar to that of the Polish population. Most of the prisoners described access to health services as “bad”, except female prisoners from a semi-open facility. In turn, satisfaction with healthcare services was most often assessed as “bad”, except for temporarily arrested men and female prisoners from a semi-open facility. Most of the prisoners had a normal body weight according to the BMI index and did not report having chronic diseases, but they did report addiction. Less than 1/5 prisoners reported using the ambulance service.

## Data Availability

Not applicable.
